# Lithium chloride ameliorates cognition dysfunction induced by sevoflurane anesthesia in rats

**DOI:** 10.1002/2211-5463.12779

**Published:** 2020-01-08

**Authors:** Yilong Wang, Xiaohu An, Xiaoqing Zhang, Jianhui Liu, Jianwei Wang, Zeyong Yang

**Affiliations:** ^1^ Department of Anesthesiology International Peace Maternity and Child Health Hospital Shanghai Jiao Tong University School of Medicine China; ^2^ Shanghai Key Laboratory of Embryo Original Diseases China; ^3^ Shanghai Municipal Key Clinical Specialty China; ^4^ Department of Anesthesiology Tongji Hospital Tongji University Shanghai China

**Keywords:** anesthesia, apoptosis, cognitive deficits, glycogen synthase kinase‐3β, lithium chloride, sevoflurane

## Abstract

Postoperative cognitive dysfunction is a common complication in elderly patients after surgeries involving anesthesia, but the underlying mechanisms are poorly understood. Lithium is a conventional treatment for bipolar disorder, which exerts a neuroprotective role in various diseases by inhibiting glycogen synthase kinase‐3β (GSK‐3β) in the brain and spinal cord. However, it is not known whether lithium chloride (LiCl) can protect against cognitive dysfunction induced by sevoflurane (SEV) anesthesia. Here, we examined the effects of LiCl on SEV‐induced cognitive dysfunction in rats and on SEV‐induced neuron apoptosis. We report that anesthesia with SEV significantly impaired memory performance, induced oxidative stress and hippocampal neuron apoptosis, and stimulated GSK‐3β activity. Treatment with LiCl ameliorated SEV‐induced cognitive disorder in rats by inhibiting the GSK‐3β/β‐catenin signaling pathway. In addition, LiCl reduced hippocampal neuron apoptosis and oxidative stress induced by SEV anesthesia. These results suggest that LiCl may have potential for development into a therapeutic agent for treatment of SEV anesthesia‐induced cognitive dysfunction.

## Graphical abstract

LiCl ameliorates sevoflurane‐induced cognitive impairment by inhibiting apoptosis and oxidative stress through suppression of the glycogen synthase kinase‐3β/β‐catenin signaling pathway, suggesting that LiCl may have potential for development into a therapeutic agent for treating sevoflurane anesthesia‐induced neurodegeneration.

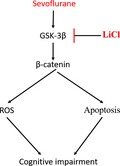

**Abbreviations**
CAT: catalase
GSK‐3β: glycogen synthase kinase‐3β
LiCl: lithium chloride
MWM: Morris water maze
POCD: postoperative cognitive dysfunction
ROS: reactive oxygen species
SOD1: superoxide dismutase 1

Anesthesia and surgery have relations with brief or long‐time deterioration in cognition condition, which was named postoperative cognitive dysfunction (POCD) 1, 2. POCD occurs more commonly in elderly people, because aging has been considered the most important risk factor in its course 3, 4.

However, not much is known about the etiology of POCD 5, 6, 7. One study showed that anesthesia with sevoflurane (SEV) might reduce neuronal survival and neurogenesis rate in the hippocampus, resulting in cognitive dysfunction and neurotoxicity in elder rats 8. In addition, an *in vitro* study demonstrated that inhaling an anesthetic might result in the activation of caspases and cell apoptosis, and also increase the production of Aβ, which can finally influence the course of Alzheimer’s disease 9. POCD is considered a severe disease, particularly in elderly people, because this condition significantly lengthens the time for rehabilitation 10, 11. POCD can prolong hospital course, hinder disease recovery, raise postoperative complications and lower the quality of life after discharge 12, 13. Yet, the current approaches to prevention and treatment for POCD are quite rare, and the treatment effect is still unsatisfactory 14, 15. Therefore, it is important to find more effective approaches to treat POCD.

Evidence suggests that cognitive impairments caused by brain trauma or surgical anesthesia are related to dysregulated signaling pathways. For example, the phosphoinositide 3‐kinase/Akt and extracellular signal‐regulated kinase 1/2 signaling pathways are inactivated, whereas glycogen synthase kinase‐3β (GSK‐3β) and neuroapoptosis or neurotoxicity are enhanced 16, 17, 18. Emerging evidence shows that SEV impairs memory consolidation in rats, possibly through inhibiting phosphorylation of GSK‐3β in the hippocampus 19. Also, our previous study already showed that SEV anesthesia caused alterations in apoptosis‐related proteins and GSK‐3β phosphorylation, and induced cognitive dysfunction in mice 20. In addition, it is reported that lithium treatment prevents apoptosis in neonatal rat hippocampus resulting from SEV exposure 21. Over 60 years, lithium chloride (LiCl), the mood stabilizer, has been used for the treatment of mental diseases, in part by directly inhibiting GSK‐3β 22, 23, 24. Although the theory of LiCl treatment is known, genetic and pharmacological research suggest that activating GSK‐3β is among the major mechanisms of LiCl 25. A recent study has suggested that LiCl probably plays a neuroprotective role in motor dysfunctions by inhibiting GSK‐3β in rats who suffered from intracerebral hemorrhage 26.

To explore the role of LiCl on the impairment of learning and memory induced by SEV, we established the rat model with cognitive impairment using SEV. Neuroapoptosis, the relevant proteins expression, memory and learning ability of rat, and the cognitive function were tested to confirm the theory of cognitive dysfunction caused by SEV anesthesia and LiCl as a potential therapeutic strategy.

## Materials and methods

### *In vivo* model

All of the *in vivo* experiments involving animal protocols were reviewed and approved by International Peace Maternity and Child Health Hospital. Prior to the experiments, Sprague Dawley rats (250 ± 10 g, 7 weeks old, male; Vital River Laboratory Animal Technology Co. Ltd., Beijing, China) were kept in a monitored 12/12 dark/light cycle lasting for 7 days and having free access to water and food. Then the rats were randomly split into three groups: the control group inhaling normal air for 6 h, the SEV group in identical conditions of 100% O_2_ with 2.5% SEV at 600 μg·kg^−1^·min^−1^ for 6 h and the LiCl + SEV group receiving 60 mg·kg^−1^ LiCl (L4408; Sigma, St Louis, MO, USA) by intraperitoneal injection twice a day before SEV exposure. After anesthesia, the rats were in recovery lasting for 7 days. The dose of LiCl at 60 mg·kg^−1^ is most effective in our pre‐experiments (Fig. S1).

### Morris water maze test

Certain investigators blinded to the experimental groups were assigned to carry out the Morris water maze (MWM) test using anymaze software (Clever Sys Inc., VA, USA). The test was set in a pool with water at a temperature of 21 ± 1 °C in which the nonpoisonous white powder was put to visualize the shape of the rats. Before the hidden platform training, rats were constrained to swim and locate the hidden platform, a rectangular channel. If the rats failed, they were gently put on the platform for 10 s to be familiar with their location. After 1 day, a circular water maze replaced the rectangular maze, and the platform was hidden 1.0 cm under the water. Rats were put into the water in different locations among the trials. All rats went through four trials for 60 s at most, during which the rats that failed to get to the platform were put on it to be familiar with the surroundings. Then we carried out the probe trial where the platform was taken out. The rats were put in the quadrant that was opposite from the target quadrant and swam for 60 s. Thereafter, the rats were trained to find a visible platform providing a black pole as a mark; rats went through four trials each day; meanwhile, the platform and start location were changed during each trial.

### Barnes maze test

The impairment of spatial learning was analyzed with the Barnes maze. The settings were composed of a white round disk (diameter: 100 cm, height: 75.5 cm from the ground) with 12 holes (diameter: 4 cm) equally placed in a circle. One escape hole was linked to the opening on the black acrylic box (13 cm × 17 cm × 7 cm) just under the disk, with the remaining holes kept open. After about 10 s, we sank the cylinder below the water surface, and the investigator controlled the experiment remotely to allow rats starting from a random location. If the rats could not go into the escape hole in 300 s, the investigator gently guided them to the hole. Before the rats returned to their cage, they were placed in the escape box for 60 s. The rats underwent four trials each day and repeated them for 5 days. The location of the hole stayed the same for all rats during the training course. The rats could memorize the location of the hole by using the materials from the room as visual marks. The rats that failed to find the hole for eight consecutive trials were kept out from this experiment. On the sixth day, the rats went through a probe test for 300 s in the maze to test whether they could memorize the position of the hole, while the box was removed from its original place. During all trials, including the probe test, we used a charge‐coupled device camera to record the whole view of the maze, and the camera was connected to the computer. The data were analyzed by matlab software (MATLAB 7.0, MathWorks, St. Louis, MO, USA), which could automatically track and analyze the escape routes.

### Measurement of intracellular reactive oxygen species

Tissues were incubated in 25 μm 2ʹ,7ʹ‐Dichlorodihydrofluorescein diacetate for about 30 min and rinsed twice using PBS. The excitation and emission wavelengths were 515 and 585 nm, respectively, and the fluorescence intensity was measured with a luminometer.

### Superoxide dismutase 1 activity assay

All reagents we used were purchased from Sigma‐Aldrich Sp. z o.o. (Poznań, Poland), which included HCl, (−) epinephrine, EDTA‐Na_2_, Na_2_CO_3_/NaHCO_3_ buffer (0.05 m, pH 10.2), ethanol and chloroform. They were used for extracting superoxide dismutase (SOD). EtOH (v/v; 3 : 5), chloroform, hemolysate and distilled water were mixed in a test tube. The mixture was then vortexed vigorously and centrifuged (3824 ***g***; 4 °C; 5 min). After SOD was extracted, adrenaline and the Na_2_CO_3_/NaHCO_3_ buffer were added and incubated for 3 min at 37 °C. The UV/VIS Lambda 40 spectrophotometer was used to analyze SOD1 activity. Setting the wavelength at 320 nm (at 30 °C), the absorbance of the materials was analyzed for 5 min. The activity of SOD1 was measured per gram of hemoglobin in red blood cell. The variation coefficient and assay sensitivity were <4% and 97%, and the specificity was 0.1 U·mL^−1^.

### Catalase activity assay

Chemical reagents were purchased from Sigma‐Aldrich. Phosphoric buffer (50 mm) was applied to dilute the hemolysate to 500‐fold. A UV/VIS Lambda 40 spectrophotometer (Perkin‐Elmer, St. Louis, MO, USA) was used to analyze catalase (CAT) activity. Setting the wavelength at 1240 nm (at 30 °C), absorbance detection of the experiment samples was conducted within 30 s. CAT activity was measured according to the calibration curve. The activity of CAT was determined per gram of hemoglobin in erythrocytes. The assay specificity and variation coefficient were 89% and < 2%, and the sensitivity was 1.71 U·mL^−1^.

### TUNEL assay

The terminal deoxynucleotidyl transferase dUTP nick end labeling (TUNEL) assay was conducted to determine whether DNA fragmentation, a hallmark of apoptosis, occurred in the region of rat hippocampus. After the sections (6 μm) embedded with paraffin were prepared, the TUNEL staining was performed with the *in situ* cell death detection kit (Thermo Fisher Scientific, St. Louis, MO, USA). A Nikon Labophot 2 microscope was used to count the total number of cells and TUNEL‐positive cells in the dentate gyrus of hippocampus at 200× magnification. DAPI stains nucleus. Cells were quantified using a microscope, and six randomly selected visual fields were assessed for every assay.

### Western blotting analysis

Cell homogenization was carried out using lysis buffer [50 mm Tris (pH 7.4), 150 mm NaCl, 1% Nonidet P‐40 (FNN0021; Thermo)], 0.5% sodium deoxycholate (D6750; Sigma) and 0.1% SDS (74255; Sigma). A 40‐μg protein aliquot of each sample was separated using SDS/PAGE (10–15%) and was electrophoretically transferred onto poly(vinylidene difluoride) membranes (IPVH00010; Millipore, Billerica, MA, USA). At room temperature, the membrane was blocked using 5% BSA for 1 h. The proteins were probed with anti‐Bax Ig (1 : 1000, 2774; Cell Signaling Technology, St. Louis, MO, USA), anti‐β‐catenin Ig (1 : 1000, 8480; Cell Signaling Technology), anti‐caspase‐3 Ig (1 : 1000, 9664; Cell Signaling Technology), anti‐JNK Ig (1 : 1000, 9257; Cell Signaling Technology), anti‐Bcl2 Ig (1 : 1000, 3498; Cell Signaling Technology), anti‐p‐JNK Ig (1 : 1000, 4668; Cell Signaling Technology), anti‐GSK‐3β Ig (1 : 1000, 12456; Cell Signaling Technology), anti‐β‐actin Ig (1 : 1000, 4970; Cell Signaling Technology) and anti‐p‐GSK‐3β Ig (1 : 1000, 5558; Cell Signaling Technology) for 12 h at 4 °C. The membranes were washed four times with TBST. A secondary antibody conjugated with HRP (1 : 10 000, 7074; Cell Signaling Technology) was used for immunoblot detection. The protein bands were visualized with a chemical luminescence reagent (Pierce, Pleasanton, CA, USA). Protein levels were determined by normalizing to the level of β‐actin.

### Statistics

Data are demonstrated as mean ± SEM. Followed by Tukey’s *post hoc* analysis, a one‐way ANOVA was applied to compare data from multiple groups. Differences were considered statistically significant when the *P* value was <0.05.

## Results

### LiCl improves SEV‐induced memory impairment in rats

**Figure 1:**
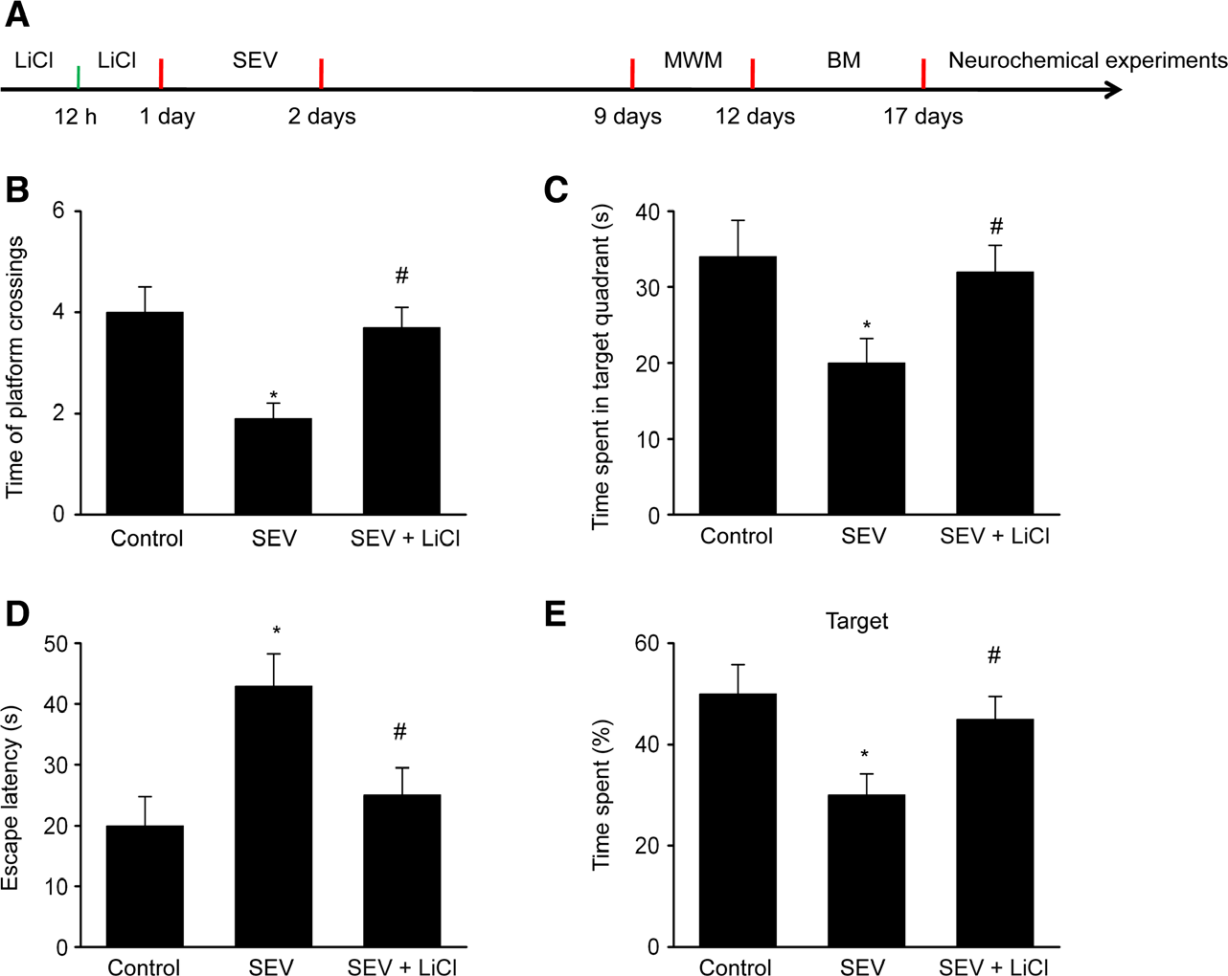
LiCl improves SEV‐induced memory impairment in rats. (A) Experimental time course. (B, C) After removing the platform on the sixth day, we counted the number of platform crossings (B) and the time spent in the target quadrant (C) within 90 s in the MWM test. (D) The latency for rat to enter a target hole in the training courses on the fifth day in BM test. (E) The percent of time spent in the target in the BM test. Data are represented as mean ± SEM; *n* = 12; one‐way ANOVA, *F* (2, 33) = 58.72; *P < 0.05 compared with control group; ^#^ *P* < 0.05 compared with SEV group.

To evaluate the memory ability and spatial learning of rats between multiple groups, the investigators made them to go through the Barnes maze test and MWM test. As presented in Fig. 1A–E, rats exposed in SEV presented cognitive impairment, which was suggested by decreased platform crossings, reduced time in the objective quadrant and prolonged escape latency. The SEV‐induced cognitive impairment was ameliorated when treated with LiCl, as presented as less escape latency, increased time in the objective quadrant and raised platform crossings.

### LiCl suppresses SEV‐induced oxidative stress in the hippocampus

**Figure 2:**
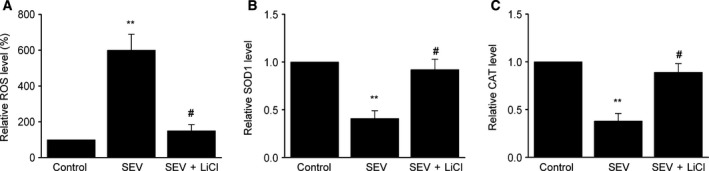
LiCl inhibits oxidative stress induced by SEV in rat hippocampus. (A) Quantification data of ROS level in the hippocampus. (B) SOD1 activity was determined by SOD1 activity colorimetric assay kit. (C) CAT activity was determined by CAT‐specific activity assay kit. Data are represented as mean ± SEM; *n* = 8; one‐way ANOVA, *F*(2, 21) = 48.92; **P < 0.01 versus control group; ^#^ *P* < 0.05 versus SEV group.

Oxidative stress is important in SEV‐induced memory deficits 26. As shown in Fig. 2A, compared with control group, reactive oxygen species (ROS) production was greatly increased in the SEV group. However, LiCl treatment reduced the level of ROS induced by SEV. Moreover, LiCl treatment markedly increased the SOD1 and CAT levels in the hippocampus (Fig. 2B,C). These results demonstrate that LiCl suppresses oxidative stress induced by SEV in rat hippocampus.

### LiCl reduces SEV‐induced apoptosis in the hippocampus

**Figure 3:**
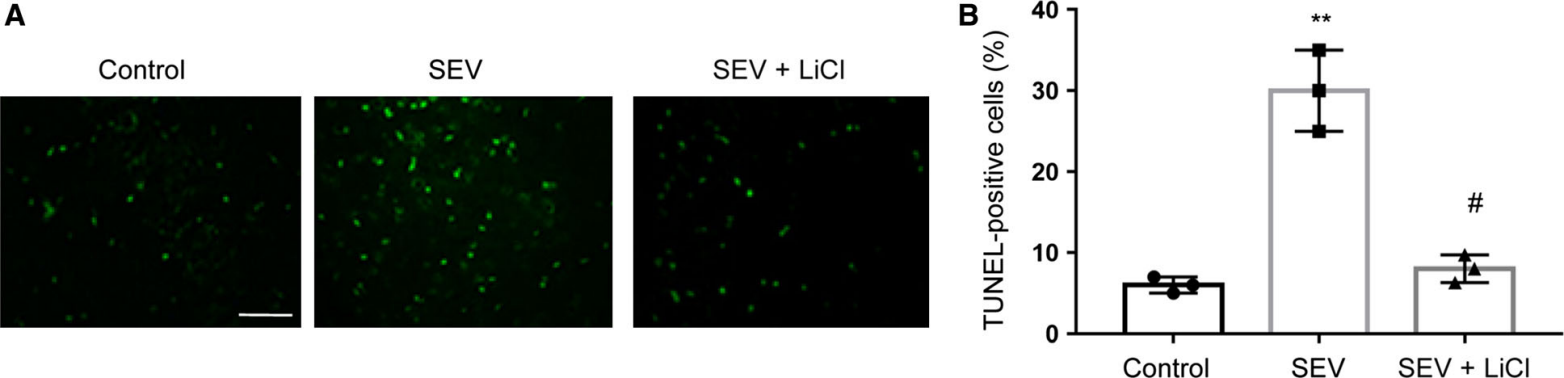
LiCl reduces apoptosis induced by SEV in rat hippocampus. (A) In each group, photomicrographs presenting TUNEL‐positive cells in rat hippocampus. (B) A chart for TUNEL‐positive cells/total number of cells ratio in different groups, and five aspects were viewed in every rat. Scale bar: 40 μm. Data are represented as mean ± SEM; *n* = 3; one‐way ANOVA, *F* (2, 6) = 55.24; **P < 0.01 compared with control group; ^#^ *P* < 0.05 compared with SEV group.

Apoptosis plays a major part in the pathological mechanisms of SEV‐induced memory impairment 27. As shown in Fig. 3A, the results from the TUNEL assay demonstrated that SEV significantly increased the number of TUNEL^+^ cells/total number of cells ratio in rat hippocampus, whereas LiCl decreased the proportion in rat hippocampus.

### LiCl decreases SEV‐induced apoptosis‐rated protein expression in rat hippocampus

**Figure 4:**
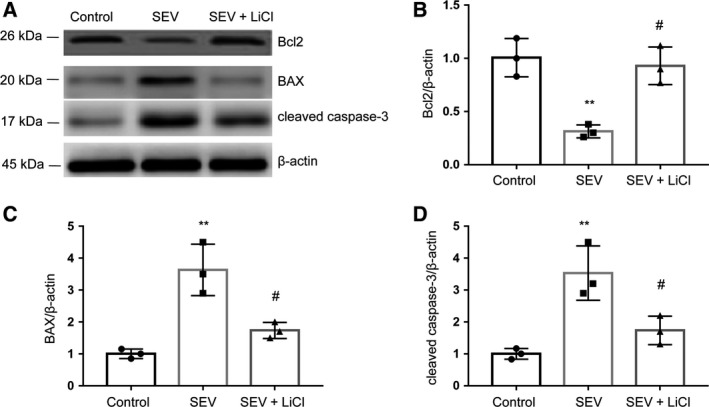
LiCl decreases SEV‐induced apoptosis‐rated proteins expression in rat hippocampus. (A–D) Immunoblots (A) and quantitative measurement of Bcl2 (B), BAX (C) and cleaved caspase‐3 (D) in rat hippocampus. Data are demonstrated as mean ± SEM; *n* = 3; one‐way ANOVA, *F* (2, 6) = 19.27; **P < 0.01 compared with control group; ^#^ *P* < 0.05 compared with SEV group.

We measured the protein expressions of Bax, Bcl‐2 and cleaved caspase‐3, which are widely considered as apoptosis indicators in cells. The results demonstrated that SEV could remarkably increase the level of Bax and cleaved caspase‐3, and reduced the level of Bcl‐2 (Fig. 3B). LiCl greatly increased the expression of Bcl‐2, reduced the expression level of Bax and cleaved caspase‐3 induced by SEV (Fig. 4A–D).

### LiCl inhibits the GSK‐3β/β‐catenin signal channel activation in the hippocampus

**Figure 5:**
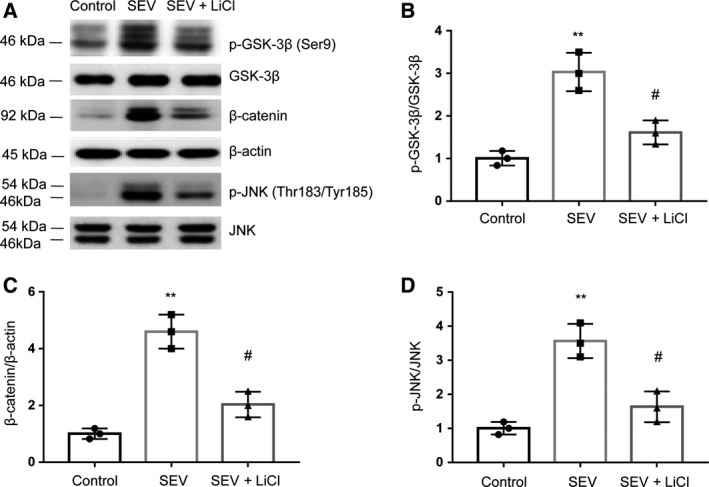
LiCl inhibits the activation of GSK‐3β/β‐catenin signal pathway in rat hippocampus. (A–D) Western blots results (A) and quantitative measurement of GSK‐3β (B), β‐catenin (C) and JNK (D) in rat hippocampus. Data are represented as mean ± SEM; *n* = 3; one‐way ANOVA, *F* (2, 6) = 31.33; **P < 0.01 compared with control group; ^#^ *P* < 0.05 compared with SEV group.

To assess the effect of LiCl on GSK‐3β/β‐catenin signal channel, which is involved in cognitive impairments, we examined the protein levels of β‐catenin, JNK and GSK‐3β. Our results show that SEV could greatly increase the phosphorylated level of GSK‐3β (Ser9) and JNK (Thr183/Tyr185) (Fig. 5A,B). LiCl significantly ameliorated the increased expression of JNK, GSK‐3β and β‐catenin, which were induced by SEV (Fig. 5C,D). These results show that LiCl can inhibit the activation process of the GSK‐3β/β‐catenin signal pathway in rat hippocampus.

## Discussion

Cognitive impairment induced by anesthesia might be the most common type in postoperative cognitive disorder, which was found to influence signal pathways dysfunction, such as activation of GSK‐3β 28, 29, 30, 31, 32, 33. This research was aimed to reveal the role of GSK‐3β inhibition on cognitive impairment induced by SEV anesthesia. We identified that GSK‐3β inhibition had a neuroprotective effect from SEV anesthesia. LiCl reduced the neuroapoptosis induced by SEV, oxidative stress and the motor dysfunction in rats via inhibiting the GSK‐3β/β‐catenin signal pathway.

GSK3β is a kinase that, on activation, leads to the Tau phosphorylation process. The SEV induces GSK3β to activate Tau phosphorylation in rat hippocampus 30. Strong evidence has demonstrated that GSK‐3β activation is involved in the excitotoxicity process of memory dysfunction in neurodegenerative diseases 34, 35. The GSK‐3β/β‐catenin signal pathway is crucial for neuronal apoptosis, and its activation accelerates hippocampal neuroapoptosis by targeting proteins related to apoptosis or interacting with other signal pathways 36, 37. In this study, significantly activated GSK‐3β was identified in the hippocampus of rats given SEV, which was consistent with other studies. Lithium could inhibit GSK3β. We found that lithium could reduce GSK3β activation, induced by SEV anesthesia, neural apoptosis and cognition impairment in rats.

The process of learning and memory appears to involve complex mechanisms that are affected by many aspects. Neural death through apoptosis can result in learning dysfunction and deficits in memory consolidation 38, 39. Cognitive impairment induced by anesthesia has been related with neuronal apoptosis caused by activated GSK‐3β in some parts of the central nervous system 40. Here, we proved that LiCl could inhibit the GSK‐3β activation in rat hippocampus and decrease neuron apoptosis and ROS production. Thus, we speculated that LiCl suppressed the GSK‐3β activation, which resulted in reduced neuron apoptosis and oxidative stress, ultimately contributing to improvement of cognitive deficits.

## Conclusions

LiCl improved the SEV‐induced cognitive impairment by inhibiting apoptosis and oxidative stress through suppression of the GSK‐3β/β‐catenin signaling pathway, and LiCl could become a therapeutic agent in treating SEV anesthesia‐induced neurodegeneration.

## Conflict of interest

The authors declare no conflict of interest.

## Author contributions

YW and ZY designed experiments. XA and XZ carried out experiments. JL and JW analyzed experimental results. YW wrote the manuscript. ZY revised the manuscript. All authors approved the final manuscript.

## Supporting information


**Fig. S1.** LiCl improves SEV‐induced memory impairment in rats. The rats were intraperitoneally injected with LiCl (30, 60, 100 mg·kg^−1^) twice a day and then treated with SEV. After removing the platform on the sixth day, we counted the number of platform crossings within 90 s in MWM. Data are represented as mean ± SEM; *n* = 10; one‐way ANOVA, *P < 0.05 compared with control group; #*P* < 0.05 compared with the SEV group.
