# Supplementary figures and images for: Lithium chloride ameliorates cognition dysfunction induced by sevoflurane anesthesia in rats
Source: FEBS Open Bio. 2020 Jan 8;10(2):251–8. doi: 10.1002/2211-5463.12779 (PMC6996326; doi:10.1002/2211-5463.12779)

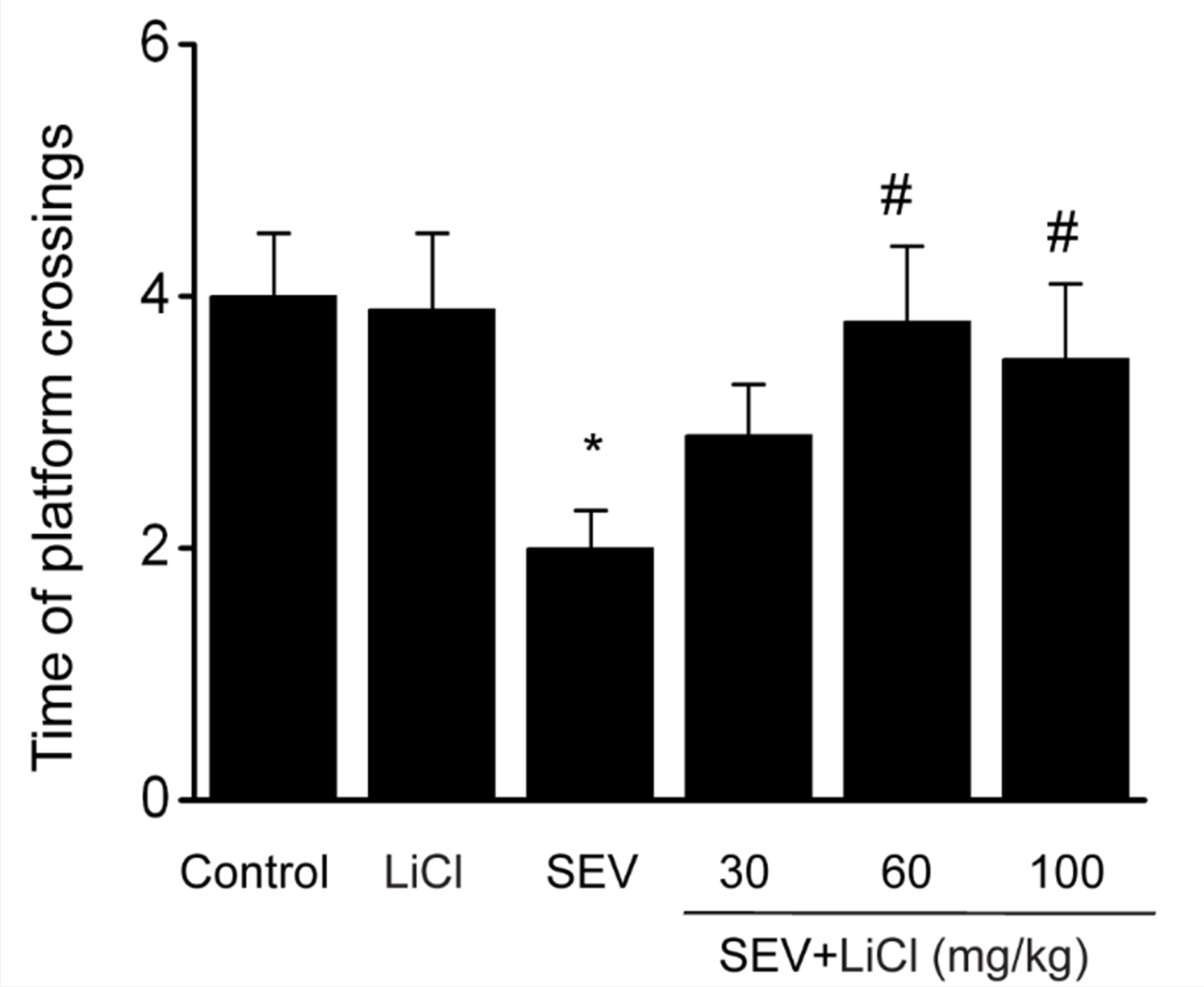

Supplement: Supplementary file 1 — Fig. S1. LiCl improves SEV‐induced memory impairment in rats. The rats were intraperitoneally injected with LiCl (30, 60, 100 mg·kg−1) twice a day and then treated with SEV. After removing the platform on the sixth day, we counted the number of platform crossings within 90 s in MWM. Data are represented as mean ± SEM; n = 10; one‐way ANOVA, *P < 0.05 compared with control group; #P < 0.05 compared with the SEV group. [file FEB4-10-251-s001.tif]
